# Probing a Coral Genome for Components of the Photoprotective Scytonemin Biosynthetic Pathway and the 2-Aminoethylphosphonate Pathway 

**DOI:** 10.3390/md11020559

**Published:** 2013-02-22

**Authors:** Eiichi Shoguchi, Makiko Tanaka, Takeshi Takeuchi, Chuya Shinzato, Nori Satoh

**Affiliations:** Marine Genomics Unit, Okinawa Institute of Science and Technology, Onna, Okinawa 904-0495, Japan; E-Mails: m.tanaka@oist.jp (M.T.); take9255837@gmail.com (T.T.); c.shinzato@oist.jp (C.S.); norisky@oist.jp (N.S.)

**Keywords:** coral genome, sunscreen, MAA, scytonemin, tyrosinase, phosphonopyruvate decarboxylase, glutamate dehydrogenase, 2-aminoethylphosphonate (AEP) pathway

## Abstract

Genome sequences of the reef-building coral, *Acropora digitifera*, have been decoded. *Acropora* inhabits an environment with intense ultraviolet exposure and hosts the photosynthetic endosymbiont, *Symbiodinium*. *Acropora* homologs of all four genes necessary for biosynthesis of the photoprotective cyanobacterial compound, shinorine, are present. Among metazoans, these genes are found only in anthozoans. To gain further evolutionary insights into biosynthesis of photoprotective compounds and associated coral proteins, we surveyed the *Acropora* genome for 18 clustered genes involved in cyanobacterial synthesis of the anti-UV compound, scytonemin, even though it had not previously been detected in corals. We identified candidates for only 6 of the 18 genes, including *tyrP*, *scyA*, and *scyB*. Therefore, it does not appear that *Acropora digitifera* can synthesize scytonemin independently. On the other hand, molecular phylogenetic analysis showed that one tyrosinase gene is an ortholog of vertebrate tyrosinase genes and that the coral homologs, *scyA* and *scyB*, are similar to bacterial metabolic genes, *phosphonopyruvate* (*ppyr*) *decarboxylase* and *glutamate dehydrogenase* (*GDH*), respectively. Further genomic searches for *ppyr* gene-related biosynthetic components indicate that the coral possesses a metabolic pathway similar to the bacterial 2-aminoethylphosphonate (AEP) biosynthetic pathway. The results suggest that *de novo* synthesis of carbon-phosphorus compounds is performed in corals.

## 1. Introduction

Reef-building corals (Class Anthozoa) typically inhabit shallow and relatively clear tropical waters; therefore, they are constantly exposed to high levels of ultraviolet radiation. Since corals are particularly susceptible to bleaching when exposed to both rising temperatures and high solar radiation [1,2], one intriguing question is how corals protect themselves against ultraviolet damage. UV-absorbing substances potentially act as photoprotective compounds. These include mycosporine-like amino acids (MAAs), scytonemin, carotenoids, and other compounds of unknown structure [3,4]. These photoprotective compounds have been isolated from various marine organisms, including corals [5,6]. However, since reef-building corals maintain symbiotic dinoflagellates, such as *Symbiodinium*, in the gastrodermal tissue layer [7,8], and since dinoflagellates can independently synthesize photoprotective compounds [9], the origins of these compounds are often uncertain [10]. 

Following the sequencing of the genome of the sea anemone (anthozoan) *Nematostella vectensis* [11], Starcevic *et al.* [12] investigated whether the *Nematostella* genome contains genes for enzymes of the shikimic acid pathway, which contributes to the biosynthesis of MAAs. They found that the *Nematostella* genome contains genes encoding aroB (dehydroquinate synthase (DHQS)) and other genes from the same pathway. The *Nematostella* genes are closely related to those of dinoflagellates, suggesting that the *Nematostella* genes were acquired via horizontal gene transfer (HGT) [12]. Recently, the genome of the hydrozoan, *Hydra magnipapillata*, was also sequenced [13], and the presence of retained genes in cnidarians, not found in the other animal genomes, has been reported [14]. 

We have now sequenced the genome of the coral, *Acropora digitifera*, using Roche 454 GS-FLX and Illumina GAIIx sequencers, obtaining approximately 110-fold coverage with whole-genome shotgun, paired-end and mate-pair methods [15]. The coral genome was estimated to be 420 Mbp in size. We identified 23,668 gene models in the coral genome; 16,434 of these are complete gene models with both start and stop codons. Approximately 93% of the coral gene models have counterparts in other metazoan genomes [15].

Recently, Balskus and Walsh [16] identified a four-gene cluster (encoding DHQS-like, *O*-MT (*O*-methyltransferase), ATP-grasp, and NRPS-like (nonribosomal peptide synthetase-like) enzymes) that is required for conversion of pentose-phosphate metabolites into shinorine (an MAA) in the cyanobacterium, *Anabaena variabilis*. We scanned the *Acropora* gene models for homologs of the shinorine gene cluster, and found that this four-gene pathway is present in both *Acropora* and *Nematostella*, but not in *Hydra* [15]. This strongly suggests that both *Acropora* and *Nematostella* can synthesize shinorine, which may be used to produce photoprotective compounds. In addition, by molecular phylogenetic analyses, we showed that the homologous putative proteins in *Acropora* had more sequence similarities to those of bacteria and dinoflagellates than to those of humans and *Drosophila* [15].

**Figure 1:**
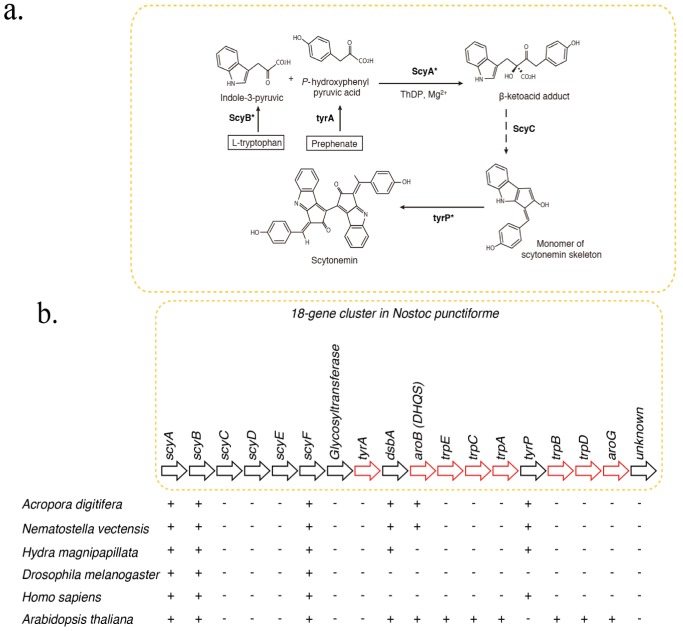
Distribution of genes associated with biosynthesis of scytonemin in cyanobacteria, cnidarians, and other metazoans. (**a**) Pathways of biosynthesis of the photoprotective molecule, scytonemin, in the cyanobacterium, *Nostoc punctiforme* [6,16]. Gene homologs encoding enzymes indicated with asterisks were identified in the *A. digitifera* genome. (**b**) Schematic showing the organization of the scytonemin gene cluster. Genes indicated by red arrows encode enzymes involved in the biosynthesis of aromatic amino acids. The presence of corresponding genes in various organisms is indicated by “+”, indicating that a TBLASTN search against *N. punctiforme* as query showed significant hits. Anthozoan genomes encode a gene homologous to *aroB*, involved in aromatic amino acid metabolism, which is not found in higher metazoans.

The indole-alkaloid, scytonemin, is a UV-blocking compound, found exclusively in cyanobacteria, and has been evaluated for biomedical applications [17]. Recently, Soule *et al.* [18,19] showed that scytonemin synthesis is controlled by an 18-gene cluster in the cyanobacterium, *Nostoc punctiforme* (Figure 1). The *Nostoc* operon includes *scyA*, *scyB*, *scyC*, *scyD*, *scyE*, *scyF*, *NpR1270* (*glycosyltransferase*), *tyrA*, *dsbA* and *aroB*. Although scytonemins have not been found in corals, the presence of symbiotic cyanobacteria in coral species has been reported [20]. Furthermore, some cyanobacteria have been implicated in coral disease [21] and the roles of microbial communities associated with coral are being discussed [22]. Therefore, in this study, we investigated whether the coral genome contains genes encoding proteins that are homologous to cyanobacterial enzymes involved in scytonemin synthesis. In relation to the homologs of *scyA*, we surveyed the *Acropora* genome for genes encoding enzymes of the 2-aminoethylphosphonate (AEP) pathway. AEP is a natural carbon-phosphorus compound, first reported by Horiguchi & Kandatsu [23]. This study will provide a basis for natural product surveys of anthozoans. 

## 2. Results and Discussion

The UV-blocking compound, scytonemin, is produced exclusively by cyanobacteria (Figure 1). The probable biosynthetic pathway has been reported [24] (Figure 1a). The scytonemin gene cluster in *Nostoc punctiforme* consists of one subcluster of genes involved in aromatic amino acid biosynthesis, but the functions of many novel genes in another subcluster are unknown [19]. The former subcluster includes *tyrA*, *dsbA*, *aroB*, *trpE*, *trpC*, *trpA*, *tyrP*, *trpB*, *trpD* and *aroG* (Figure 1b, red arrows). The latter includes *scyA*, *scyB*, *scyC*, *scyD*, *scyE*, and *scyF* (Figure 1b, black arrows).

Screening of the *A. digitifera* genome via BLAST and domain structure comparisons led to the identification of candidates for six of the 18 genes involved in scytonemin synthesis: *scyA*, *scyB*, *scyF*, *dsbA*, *aroB*, and *tyrP* (Figure 1b). Analysis of *aroB* (*DHQS*) in a previous study identified an *aroB* homolog in the *Acropora* genome [15]. Molecular phylogenetic analyses group the *aroB-like* sequences of *Acropora* and *Nematostella* with those of several dinoflagellates,ss, consistent with the possibility that the *aroB-like* genes of cnidarians originated by horizontal transfer from dinoflagellates [12]. Here we describe results of molecular phylogenetic analyses of *scyA*, *scyB*, *dsbA*, and *tyrP.* Detailed analyses of *scyF* homologs were not performed for reasons that will be explained subsequently (See Section 2.3).

### 2.1. scyA (TPP-Dependent Enzyme)

**Figure 2:**
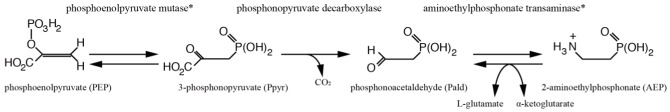
Metabolic pathways unique among metazoans and found in corals. The 2-aminoethylphosphonate (AEP) biosynthetic pathway was first discovered in *Tetrahymena pyriformis*. Phosphoenolpyruvate decarboxylase, shown in Table 1, is uncommon in metazoans. Homologs of the other two enzyme genes involved, indicated by asterisks, are also found in coral; see Table 2 for details.

*scyA* encodes a TPP (thiamine pyrophosphate)-dependent enzyme [25], a protein similar to human 2-hydroxyacyl-CoA lyase, which has close homologs in a variety of organisms, including *Drosophila* and *Arabidopsis* (Figure 1b; Table 1). It is also similar to acetolactate synthase which is found in plants and micro-organisms. Both 2-hydroxyacyl-CoA lyase and acetolactate synthase are involved in synthesis of the essential amino acids, valine, leucine, and isoleucine [26]. Biosynthesis of 2-aminoethylphosphonate (AEP) from phosphoenolpyruvate (PEP) requires just three enzymes: PEP mutase, phosphonopyruvate decarboxylase, and AEP transaminase, collectively known as the AEP biosynthetic pathway [27] (Figure 2; See Section 2.6). Phosphonopyruvate (ppyr) decarboxylase is also similar to both 2-hydroxyacyl-CoA lyase and acetolactate synthase. 

**Table 1:** Putative enzyme genes in the *Acropora digitifera* genome that are similar to enzymes involved in biosynthesis of the cyanobacterial sunscreen, scytonemin.

Gene name	Gene model ID	Intron number	All PFAM domains (in order) *	corresponding to ESTs	scaffold	References
*phosphonopyruvate decarboxylase*	aug_v2a.20271	6	TPP_enzyme_N, TPP_enzyme_C	+	12471	Figure S1
*2-hydroxyacyl-CoA lyase 1*	aug_v2a.06817	13	TPP_enzyme_C	−	2544	Figure S1
*glutamate dehydrogenase1-1 (gdh1-1)*	aug_v2a.22675	0	ELFV_dehydrog_N, ELFV_dehydrog	+	15779	Figure S2
*glutamate dehydrogenase1-2 (gdh1-2)*	aug_v2a.23483	1	ELFV_dehydrog_N, ELFV_dehydrog	+	16875	Figure S2
*glutamate dehydrogenase2-1 (gdh2-1)*	aug_v2a.13667	6	ELFV_dehydrog_N, ELFV_dehydrog	+	5605	Figure S2
*glutamate dehydrogenase2-2* *(gdh2-2)*	aug_v2a.16277	7	ELFV_dehydrog_N, ELFV_dehydrog	−	7525	Figure S2
*DSBA domain containing gene-1*	aug_v2a.12085	21	Dynein_Heavy, DSBA, DSBA	+	4763	Figure S3
*DHQS-like* (*aroB-like*)	aug_v2a.14548	2	DHQ_synthase	+	6105	[15]
*TyrP1*	aug_v2a.08070	2	TSP_1, TSP_1, TSP_1, TSP_1, Tyrosinase	+	3066	Figure S4
*TyrP2*	aug_v2a.10437	12	Tyrosinase	+	4001	Figure S4

* Search parameters: *E*-value of 1.0.

Molecular phylogenetic analysis showed that two *Acropora* proteins containing a TPP enzyme domain were separated into two clades, one containing PEP decarboxylase, with orthology to the *Bacteroides fragilis* enzyme and the other, 2-hydroxyacyl-CoA lyase, with orthology to the human protein (Table 1; Figure S1). Both enzymes have *Nematostella* counterparts, and these were closely related to each other (Figure S1). In contrast, the latter group formed a clade that includes *Homo*, *Drosophila*, and *Arabidopsis* orthologs. PEP decarboxylase was not found in other metazoan genomes. The *Acropora PEP decarboxylase* gene has six introns and was located at the 5′ terminus of scaffold 12471. Its neighbor was a gene for an ephrin-like protein, which belongs to the tyrosine kinase receptor subfamily. mRNA corresponding to *ppyr decarboxylase*, but not *hydroxyacyl-CoA lyase*, was present in EST databases (Table 1). The gene for acetolactate synthase was not found. Neither of the two *Acropora* genes formed a clade with *scyA* of the cyanobacteria, *Nostoc* and *Nodularia*.

### 2.2. scyB (GDH Subfamly)

*scyB* encodes a protein that resembles glutamate dehydrogenase (GDH) [17]. GDH enzymes are divided into four classes [28,29]. Searches for GDH genes in the *Acropora* genome revealed four genes, *gdh-1-1*, *-1-2*, *-2-1*, and *-2-2* (Table 1). Molecular phylogenetic analysis indicated that *gdh-2-1* and *gdh-2-2* form a clade with *Nematostella* and *Hydra* orthologs (Figure S2). This clade also includes orthologs of *Drosophila* and *Homo*, suggesting that *gdh-2-1* and *gdh-2-2* encode metazoan GDH. The presence of *gdh-2-1* and *gdh-2-2* in one clade implies that they were duplicated within the lineage (Table 1).

On the other hand, *gdh-1-1* and *gdh-1-2* form another clade with the corresponding *Nematostella* genes (Figure S2). This group includes bacterial and *Arabidopsis* genes, but not those of metazoans (Figure S2). All trees (Bayesian inference, Neighbor joining, and Maximum likelihood) supported the clade (Figure S2). *gdh-1-1* has no introns while *gdh-1-2* has one. The expression of *gdh-1-1* was confirmed in the EST database. *gdh-1-2* was located at the 5′ terminus of scaffold 16875 and the neighboring gene is similar to human caseinolytic peptidase B, a hexameric chaperone. This analysis indicates that corals have two GDH class 1 and two GDH class 2 enzymes. Because GDH class 1 has not been found in metazoans [29], corals may have unknown GDH metabolic pathways.

### 2.3. scyF (NHL Repeat Containing)

NHL is a conserved structural motif present in a large family of growth regulators. Many NHL-containing proteins also possess additional domains, e.g., RING fingers, B-box zinc fingers, and coiled-coil motifs. According to structural model analysis, the NHL domain-containing genes could be involved in protein−protein interactions and/or protein-nucleic acid interactions [30]. *scyF* encodes a protein that contains an NHL repeat (Ncl-1, HT2A and Lin-41), which is defined by amino acid sequence similarities to Ncl-1, HT2A, and Lin-41 proteins [30].

Most animal and plant genomes contain *scyF*-like genes (Figure 1b). A Pfam domain search of the NHL domain revealed that the *Acropora* genome contains 107 genes encoding NHL-containing proteins. In addition, the three *Acropora* genes most similar to *Nostoc scyF*, aug-v2a.11071, aug-v2a.01011, and aug-v2a.06686, included other domains such Filamin, SGL, and zf-B Box. Therefore, it was difficult to clarify the relationship among NHL-repeat-containing genes. Only three genes encode proteins with one NHL repeat each. Some of these may be members of novel metabolic pathways. 

### 2.4. dsbA

DsbA (disulfide bond A) is a subfamily of the thioredoxin family [31,32]. Efficient, correct folding of bacterial disulfide-bonded proteins *in vivo* is dependent upon a class of periplasmic oxidoreductase proteins called DsbA. The bacterial protein-folding factor DsbA is the most oxidizing member of the thioredoxin family. 

*dsbA* genes with high similarities to *Nostoc dsbA* have been identified in each of the cnidarians (*A. digitifera*, *Nematostella vectensis* and *Hydra magnipapillata*) and in *Trichoplax* (Phylum Placozoa), but are not found in *Drosophila* and *Homo* (Table 1). Metazoan *dsbA* genes have greatly diverged from bacterial *DsbA* genes; therefore, it was difficult to align the sequences. Such low similarities may be due to selenoproteins, in which it is difficult to predict the open reading frame [33]. By domain search, we found three candidates, aug-v2a.12085, aug-v2a.05997, and aug-v2a.00764 in the *Acropora* genome. However, the gene models, aug-v2a.05997 and aug-v2a.00764, were likely partial, and were excluded from further analyses. These models may be artifacts of insufficient assembly or inaccurate gene prediction. The four cnidarian *dsbA* sequences formed discrete clades in molecular phylogenetic analyses (Figure S3), suggesting diversification of these genes in the cnidarian lineage. In addition, *DSBA domain-containing gene-1* was positioned in a subgroup different from the cyanobacterium *dsbA*.

### 2.5. TyrP

TyrP (Tyrosinase-related Protein) has a well-established role in melanin biosynthesis in mammals, and is involved in several biological functions [34]. We found six candidate tyrosinases, but four of them were partial sequences. Therefore, we used only the two complete candidates for molecular phylogenetic analysis. Interestingly, TyrP 1 forms a clade with its vertebrate equivalents (Figure S4), although we could not find any *Nematostella* and *Hydra* orthologs in this clade (Figure S4). On the other hand, TyrP 2 is a member of a group that included the tyrosinase-related proteins of cnidarians (Figure S4). No *Acropora* tyrosinase genes form a clade with cyanobacterium TyrP, but further studies will be needed to understand the relationships of the four unknown, partial genes.

### 2.6. Genes for AEP Pathway

Because it has been reported that PEP decarboxylase is an enzyme for one of three steps in the AEP biosynthetic pathway in protists and bacteria [35], we surveyed homologs of enzyme genes for the other two steps. Interestingly, we found candidate genes for phosphoenolpyruvate mutase and aminoethylphosphonate transaminase (Table 2; Figures S5 and S6). Our gene survey suggests that *Acropora digitifera* has a complete AEP biosynthetic pathway from phosphoenolpyruvate (PEP) (Figure 2, Figures S1, S5 and S6), which is the shortest known pathway for construction of natural phosphonate [35]. Therefore, corals may be important producers of carbon-phosphorus compounds in marine ecosystems.

It is possible that reported draft genome sequences of metazoans could include sequences from other organisms, resulting from contamination. However, the coral *A. digitifera* genome sequences from the purified sperm genomic DNA of one individual did not contain contaminated sequences [15]. The following observations indicate that all of the annotated genes in this study are encoded by the *A. digitifera* genome: (1) Orthologs of these genes, which formed a clade in molecular phylogenetic analysis, were found in *Nematostella*; (2) Expression of most genes was confirmed by embryonic transcriptome analysis; and (3) Some of the gene orders, including annotated genes, were conserved between *A. digitifera* and *N. vectensis*.

**Table 2:** Orthologs of genes for the AEP biosynthetic pathway in the *Acropora digitifera* genome.

Gene name	Gene model ID	Intron number	All PFAM domains (in order) *	corresponding to ESTs	scaffold	References
*phosphoenolpyruvate mutase*	aug_v2a.19072	7	PEP_mutase	+	11028	Figure S5
*2-aminoethylphosphonate transaminase*	aug_v2a.21804	4	−	+	14440	Figure S6

* Search parameters: *E*-value of 1.0.

## 3. Experimental Section

### 3.1. Gene Search

We used two methods to search the *A. digitifera* database [36,37] for genes encoding components of the scytonemin biosynthetic pathway. First, BLAST searches with cyanobacterial protein sequences as queries (BLASTP) were used to probe *A. digitifera* gene models for putative orthologs. Genome sequences of *Nematostella vectensis* [11], *Hydra magnipapillata* [13], *Drosophila melanogaster* [38], *Homo sapiens* [39], and *Arabidopsis thaliana* [40] were also surveyed. In addition, several bacteria genes and eukaryotic genes with high similarity to *A. digitifera* models were retrieved from the NCBI genome database [41] for molecular phylogenetic analysis. The second method was the characterization of specific protein domains. To screen and identify protein domains in the gene models, we used the Pfam database [42], which contains 11,912 conserved domains using HMMER (hmmer3) [43]. In order to avoid eliminating cnidarian- or coral-specific domains, we first used an *E*-value cutoff of 10^−3^, as previously suggested [44] and subsequently an *E*-value cutoff of 1.

### 3.2. Molecular Phylogenetic Analysis

Amino acid sequences found in gene searches were aligned using ClustalX [45] with default parameters. Gaps and ambiguous areas were excluded manually, using Se-Al v2.0 [46]. For Bayesian inference analysis, the alignment datasets were analyzed using PhyloBayes 3.3 [47] with the site heterogeneous mixture CAT model and two independent Markov chains. Phylogenetic trees were constructed by Neighbor-Joining (NJ). Calculations of the NJ bootstrap value (1000 trials) were made using ClustalX, and tree constructions were performed in SeaView [48] or Njplot [49]. Maximum likelihood analyses employed TREEFINDER version October 2008 [50] and Aminosan [51]. The bootstrap value was calculated using 100 trials.

## 4. Conclusion

We have previously identified environmental response genes in corals. These included genes unique to metazoans, such as fluorescent proteins [52] and enzymes involved in shinorine synthesis [15]. The present gene survey does not support the hypothesis that *A. digitifera* can synthesize scytonemin independently. Although the *A. digitifera* genome contains homologs of several genes that function in scytonemin synthesis in *Nostoc*, these genes may have acquired new functions in *Acropora* that remain to be elucidated. The homologs of *scyA* and *scyB*, *ppyr decarboxylase*, *gdh-1-1*, *and gdh-1-2* are similar to genes involved in general bacterial metabolic pathways. Our genome-wide surveys for genes of enzymes involved in synthesis of photoprotective compounds indicate that corals retain genes for some enzymes not found in *Homo* and *Drosophila*. Therefore, it is likely that not only marine bacteria, but also marine invertebrates produce many unknown natural compounds, as suggested by the presence of the AEP pathway. Genomic surveys will undoubtedly provide more clues regarding natural product synthesis.

## Supplementary Files

Supplementary File 1 Supplementary Information (PDF, 948 KB)

## References

[1] Hoegh-Guldberg O., Mumby P.J., Hooten A.J., Steneck R.S., Greenfield P., Gomez E., Harvell C.D., Sale P.F., Edwards A.J., Caldeira K. (2007). Coral reefs under rapid climate change and ocean acidification. Science.

[2] Weis V.M. (2008). Cellular mechanisms of Cnidarian bleaching: Stress causes the collapse of symbiosis. J.Exp. Biol..

[3] Shick J.M., Romaine-Lioud S., Ferrier-Pages C., Gattuso J.P. (1999). Ultraviolet-B radiation stimulates shikimate pathway-dependent accumulation of mycosporine-like amino acids in the coral *Stylophora pistillata* despite decreases in its population of symbiotic dinoflagellates. Limnol. Oceanogr..

[4] Reef R., Dunn S., Levy O., Dove S., Shemesh E., Brickner I., Leggat W., Hoegh-Guldberg O. (2009). Photoreactivation is the main repair pathway for UV-induced DNA damage in coral planulae. J. Exp. Biol..

[5] Gordon B.R., Leggat W. (2010). Symbiodinium-invertebrate symbioses and the role of metabolomics. Mar. Drugs.

[6] Rastogi R.P., Richa, Sinha R.P., Singh S.P., Hader D.-P. (2010). Photoprotective compounds from marine organisms. J. Ind. Microbiol. Biotechnol..

[7] Dubinsky Z. (1990). Coral Reef.

[8] Stat M., Morris E., Gates R.D. (2008). Functional diversity in coral-dinoflagellate symbiosis. Proc. Natl. Acad. Sci. USA.

[9] Banaszak A.T., LaJeunesse T.C., Trench R.K. (2000). The synthesis of mycosporine-like amino acids (MAAs) by cultured, symbiotic dinoflagellates. J. Exp. Mar. Biol. Ecol..

[10] Villarreal-Chiu J.F., Quinn J.P., McGrath J.W. (2012). The genes and enzymes of phosphonate metabolism by bacteria, and their distribution in the marine environment. Front. Microbiol..

[11] Putnam N.H., Srivastava M., Hellsten U., Dirks B., Chapman J., Salamov A., Terry A., Shapiro H., Lindquist E., Kapitonov V.V. (2007). Sea anemone genome reveals ancestral eumetazoan gene repertoire and genomic organization. Science.

[12] Starcevic A., Akthar S., Dunlap W.C., Shick J.M., Hranueli D., Cullum J., Long P.F. (2008). Enzymes of the shikimic acid pathway encoded in the genome of a basal metazoan, *Nematostella vectensis*, have microbial origins. Proc. Natl. Acad. Sci. USA.

[13] Chapman J.A., Kirkness E.F., Simakov O., Hampson S.E., Mitros T., Weinmaier T., Rattei T., Balasubramanian P.G., Borman J., Busam D. (2010). The dynamic genome of *Hydra*. Nature.

[14] Forêt S., Knack B., Houliston E., Momose T., Manuel M., Quéinnec E., Hayward D.C., Ball E.E., Miller D.J. (2010). New tricks with old genes: The genetic bases of novel cnidarian traits. Trends Genet..

[15] Shinzato C., Shoguchi E., Kawashima T., Hamada M., Hisata K. (2011). Using the *Acropora*
*digitifera* genome to understand coral responses to environmental change. Nature.

[16] Balskus E.P., Walsh C.T. (2010). The genetic and molecular basis for sunscreen biosynthesis in cyanobacteria. Science.

[17] Gao Q., Garcia-Pichel F. (2011). Microbial ultraviolet sunscreens. Nat. Rev. Microbiol..

[18] Soule T., Stout V., Swingley W.D., Meeks J.C., Garcia-Pichel F. (2007). Molecular genetics and genomic analysis of scytonemin biosynthesis in *Nostoc punctiforme* ATCC 29133. J. Bacteriol..

[19] Soule T., Palmer K., Gao Q.J., Potrafka R.M., Stout V., Garcia-Pichel F. (2009). A comparative genomics approach to understanding the biosynthesis of the sunscreen scytonemin in cyanobacteria. BMC Genomics.

[20] Lesser M.P., Mazel C.H., Gorbunov M.Y., Falkowski P.G. (2004). Discovery of symbiotic nitrogen-fixing cyanobacteria in corals. Science.

[21] Gantar M., Kaczmarsky L.T., Stanic D., Miller A.W., Richardson L.L. (2011). Antibacterial activity of marine and black band disease cyanobacteria against coral-associated bacteria. Mar. Drugs.

[22] Sunagawa S., DeSantis T.Z., Piceno Y.M., Brodie E.L., DeSalvo M.K., Voolstra C.R., Weil E., Andersen G.L., Medina M. (2009). Bacterial diversity and White Plague Disease-associated community changes in the Caribbean coral *Montastraea faveolata*. ISME J..

[23] Horiguchi M., Kandatsu M. (1959). Isolation of 2-aminoethane phosphonic acid from rumen protozoa. Nature.

[24] Balskus E.P., Walsh C.T. (2008). Investigating the initial steps in the biosynthesis of cyanobacterial sunscreen scytonemin. J. Am. Chem. Soc..

[25] Costelloe S.J., Ward J.M., Dalby P.A. (2008). Evolutionary analysis of the TPP-dependent enzyme family. J. Mol. Evol..

[26] McCourt J.A., Duggleby R.G. (2006). Acetohydroxyacid synthase and its role in the biosynthetic pathway for branched-chain amino acids. Amino Acids.

[27] Zhang G., Dai J., Lu Z., Dunaway-Mariano D. (2003). The phosphonopyruvate decarboxylase from *Bacteroides fragilis*. J. Biol. Chem..

[28] Miñambres B., Olivera E.R., Jensen R.A., Luengo J.M. (2000). A new class of glutamate dehydrogenases (GDH). Biochemical and genetic characterization of the first member, the AMP-requiring NAD-specific GDH of *Streptomyces clavuligerus*. J. Biol. Chem..

[29] Andersson J.O., Roger A.J. (2003). Evolution of glutamate dehydrogenase genes: Evidence for lateral gene transfer within and between prokaryotes and eukaryotes. BMC Evol. Biol..

[30] Slack F.J., Ruvkun G. (1998). A novel repeat domain that is often associated with RING finger and B-box motifs. Trends Biochem. Sci..

[31] Bardwell J.C.A., Mcgovern K., Beckwith J. (1991). Identification of a protein required for disulfide bond formation *in vivo*. Cell.

[32] Hu S.H., Peek J.A., Rattigan E., Taylor R.K., Martin J.L. (1997). Structure of TcpG, the DsbA protein folding catalyst from *Vibrio cholerae*. J. Mol. Biol..

[33] Jiang L., Liu Q., Ni J. (2010). *In silico* identification of the sea squirt selenoproteome. BMC Genomics.

[34] Halaouli S., Asther M., Sigoillot J.C., Hamdi M., Lomascolo A. (2006). Fungal tyrosinases: New prospects in molecular characteristics, bioengineering and biotechnological applications. J. Appl. Microbiol..

[35] Metcalf W.W., van der Donk W.A. (2009). Biosynthesis of phosphonic and phosphinic acid natural products. Annu. Rev. Biochem..

[36] Koyanagi R., Takeuchi T., Hisata K., Gyoja F., Shoguchi E., Satoh N., Kawashima T.  (2013). An integrated genome viewer for community-based annotation of genomes. Zool. Sci..

[37] Oist Marine Genomics Unit Genome Project. http://marinegenomics.oist.jp/acropora_digitifera.

[38] Adams M.D., Celniker S.E., Holt R.A., Evans C.A., Gocayne J.D., Amanatides P.G., Scherer S.E., Li P.W., Hoskins R.A., Galle R.F. (2000). The genome sequence of *Drosophila melanogaster*. Science.

[39] Lander E.S., Linton L.M., Birren B., Nusbaum C., Zody M.C., Baldwin J., Devon K., Dewar K., Doyle M., FitzHugh W. (2001). Initial sequencing and analysis of the human genome. Nature.

[40] Arabidopsis Genome Initiative (2000). Analysis of the genome sequence of the flowering plant *Arabidopsis thaliana*. Nature.

[41] National Center for Biotechnology Information. http://www.ncbi.nlm.nih.gov/guide/.

[42] Finn R.D., Mistry J., Schuster-Bockler B., Griffiths-Jones S., Hollich V., Lassmann T., Moxon S., Marshall M., Khanna A., Durbin R. (2006). Pfam: Clans, web tools and services. Nucl. Acids Res..

[43] Eddy S.R. (1998). Profile hidden Markov models. Bioinformatics.

[44] Kawashima T., Kawashima S., Tanaka C., Murai M., Yoneda M., Putnam N.H., Rokhsar D.S., Kanehisa M., Satoh N., Wada H. (2009). Domain shuffling and the evolution of vertebrates. Genome Res..

[45] Larkin M.A., Blackshields G., Brown N.P., Chenna R., McGettigan P.A., McWilliam H., Valentin F., Wallace I.M., Wilm A., Lopez R. (2007). Clustal W and clustal X version 2.0. Bioinformatics.

[46] Castresana J. (2000). Selection of conserved blocks from multiple alignments for their use in phylogenetic analysis. Mol. Biol. Evol..

[47] Lartillot N., Philippe H. (2004). A Bayesian mixture model for across-site heterogeneities in the amino-acid replacement process. Mol. Biol. Evol..

[48] Gouy M., Guindon S., Gascuel O. (2010). SeaView Version 4: A multiplatform graphical user interface for sequence alignment and phylogenetic tree building. Mol. Biol. Evol..

[49] Perriere G., Gouy M. (1996). WWW-Query: An on-line retrieval system for biological sequence banks. Biochimie.

[50] Jobb G., von Haeseler A., Strimmer K. (2004). TREEFINDER: A powerful graphical analysis environment for molecular phylogenetics. BMC Evol. Biol..

[51] Tanabe A.S. (2011). Kakusan4 and Aminosan: Two programs for comparing nonpartitioned, proportional and separate models for combined molecular phylogenetic analyses of multilocus sequence data. Mol. Ecol. Resour..

[52] Shinzato C., Shoguchi E., Tanaka M., Satoh N. (2012). Fluorescent protein candidate genes in the coral *Acropora digitifera* genome. Zool. Sci..

