# Supplementary material for: Probing a Coral Genome for Components of the Photoprotective Scytonemin Biosynthetic Pathway and the 2-Aminoethylphosphonate Pathway
Source: Mar Drugs. 2013 Feb 22;11(2):559–70. doi: 10.3390/md11020559 (PMC3640398; doi:10.3390/md11020559)

Supplementary Information

Figure S1. (**a**) Molecular phylogenetic tree of thiamine pyrophosphate (TPP) domain-containing enzymes, including scyA, phosphonolpyruvate decarboxylase, and 2-hydroxyacyl-CoA lyase, constructed using the Bayesian inference (BI) method. 2-hydroxyacyl-CoA lyase of *A. digitifera* forms a clade with the *N. vectensis* homolog. No thiamine pyrophosphate (TPP) domain-containing genes in Anthozoa form a clade with scyA (GI: 186681744) of cyanobacteria. Numbers at the nodes indicate a BI posterior probability greater than 0.5. The scale bar represents 0.5 expected substitutions per site in the aligned region. Species names are followed by NCBI gene ID numbers. For *N. vectensis*, protein ID numbers from the JGI genome browser are shown. The highlighted B indicates bacterial proteins; E indicates eukaryotic proteins; and a circled M denotes metazoan proteins. Putative proteins of *A. digitifera* are shown in red, *Nematostella* proteins in blue, and *Hydra* in green. (**b**) Molecular phylogenetic tree of thiamine pyrophosphate (TPP) domain-containing enzymes, including scyA and 2-hydroxyacyl-CoA lyase, constructed using the neighbor-joining (NJ) method. Both enzymes of *A. digitifera* form a clade with *N. vectensis* homologs. Numbers at the nodes indicate bootstrap supports greater than 50% (NJ, left; ML, right). The scale bar represents 0.1 expected substitutions per site in the aligned region. Species names are followed by NCBI gene ID numbers. For *N. vectensis*, protein ID numbers from the JGI genome browser are shown. The highlighted B indicates bacterial proteins; E indicates eukaryotic proteins; and a circled M denotes metazoan proteins. Putative proteins of *A. digitifera* are shown in red, *Nematostella* proteins in blue and *Hydra* in green.

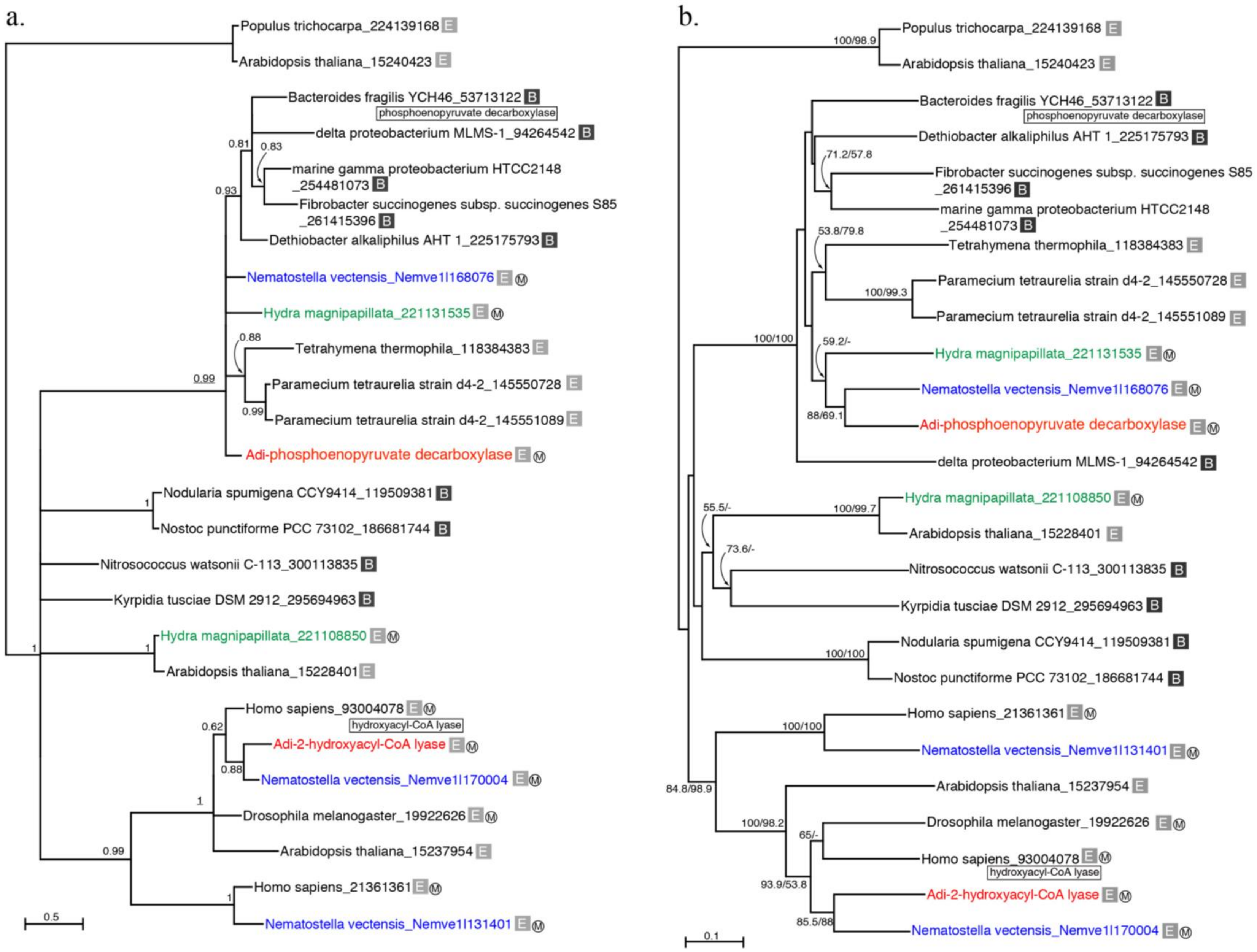

a.
Populus trichocarpa_224139168 E
Arabidopsis thaliana_15240423 E
Bacteroides fragilis YCH46_53713122 B
phosphoenopyruvate decarboxylase
delta proteobacterium MLMS-1_94264542 B
0.81
0.83
marine gamma proteobacterium HTCC2148 _254481073 B
0.93
Fibrobacter succinogenes subsp. succinogenes S85 _261415396 B
Dethiobacter alkaliphilus AHT 1_225175793 B
Nematostella vectensis_Nemve1|168076 E M
Hydra magnipapillata_221131535 E M
0.88
Tetrahymena thermophila_118384383 E
0.99
Paramecium tetraurelia strain d4-2_145550728 E
0.99
Paramecium tetraurelia strain d4-2_145551089 E
Adi-phosphoenopyruvate decarboxylase E M
1
Nodularia spumigena CCY9414_119509381 B
Nostoc punctiforme PCC 73102_186681744 B
Nitrosococcus watsonii C-113_300113835 B
Kyrpidia tusciae DSM 2912_295694963 B
1
1
Hydra magnipapillata_221108850 E M
Arabidopsis thaliana_15228401 E
Homo sapiens_93004078 E M
0.62
hydroxyacyl-CoA lyase
Adi-2-hydroxyacyl-CoA lyase E M
0.88
Nematostella vectensis_Nemve1|170004 E M
1
Drosophila melanogaster_19922626 E M
0.99
Arabidopsis thaliana_15237954 E
Homo sapiens_21361361 E M
1
Nematostella vectensis_Nemve1|131401 E M
0.5
b.
100/98.9
Populus trichocarpa_224139168 E
Arabidopsis thaliana_15240423 E
Bacteroides fragilis YCH46_53713122 B
phosphoenopyruvate decarboxylase
Dethiobacter alkaliphilus AHT 1_225175793 B
71.2/57.8
Fibrobacter succinogenes subsp. succinogenes S85 _261415396 B
marine gamma proteobacterium HTCC2148 _254481073 B
53.8/79.8
Tetrahymena thermophila_118384383 E
100/99.3
Paramecium tetraurelia strain d4-2_145550728 E
Paramecium tetraurelia strain d4-2_145551089 E
100/100
59.2/-
Hydra magnipapillata_221131535 E M
Nematostella vectensis_Nemve1|168076 E M
88/69.1
Adi-phosphoenopyruvate decarboxylase E M
delta proteobacterium MLMS-1_94264542 B
55.5/-
100/99.7
Hydra magnipapillata_221108850 E M
Arabidopsis thaliana_15228401 E
73.6/-
Nitrosococcus watsonii C-113_300113835 B
Kyrpidia tusciae DSM 2912_295694963 B
100/100
Nodularia spumigena CCY9414_119509381 B
Nostoc punctiforme PCC 73102_186681744 B
100/100
Homo sapiens_21361361 E M
Nematostella vectensis_Nemve1|131401 E M
84.8/98.9
Arabidopsis thaliana_15237954 E
100/98.2
Drosophila melanogaster_19922626 E M
65/-
Homo sapiens_93004078 E M
hydroxyacyl-CoA lyase
93.9/53.8
Adi-2-hydroxyacyl-CoA lyase E M
85.5/88
Nematostella vectensis_Nemve1|170004 E M
0.1

Figure S2. (**a**) BI molecular phylogenetic tree of homologs of glutamate dehydrogenase (GDH), including scyB. The *A. digitifera* genome contains four candidates; pairwise (1-1 and 1-2, and 2-1 and 2-2), these form clades with *Nematostella* and/or *Hydra*. No *A. digitifera* ortholog to cyanobacterium scyB (GI: 186681743) was found. Interestingly, two of the anthozoan glutamate dehydrogenases, including Adi-gdh-1-1 and Adi-gdh-1-2, form a clade with bacterial GDH. Details for gene ID, number, and scale bars are shown in Figure S1a. The highlighted B indicates bacterial proteins; E indicates eukaryotic proteins; and a circled M denotes metazoan proteins. Putative proteins *in A. digitifera* are shown in red, *Nematostella* proteins in blue, and *Hydra* in green. (**b**) NJ molecular phylogenetic tree of homologs of glutamate dehydrogenase (GDH), including scyB. The *A. digitifera* genome contains four candidates; pairwise (1-1 and 1-2, and 2-1 and 2-2), these form clades with *Nematostella* and *Hydra*. No *A. digitifera* ortholog to cyanobacterium scyB (GI: 186681743) was found. Interestingly, five of the anthozoan glutamate dehydrogenases, including Adi-gdh-1-1 and Adi-gdh-1-2, form a clade with bacterial GDH. The details for gene ID, number, and scale bars are shown in Figure S1b.

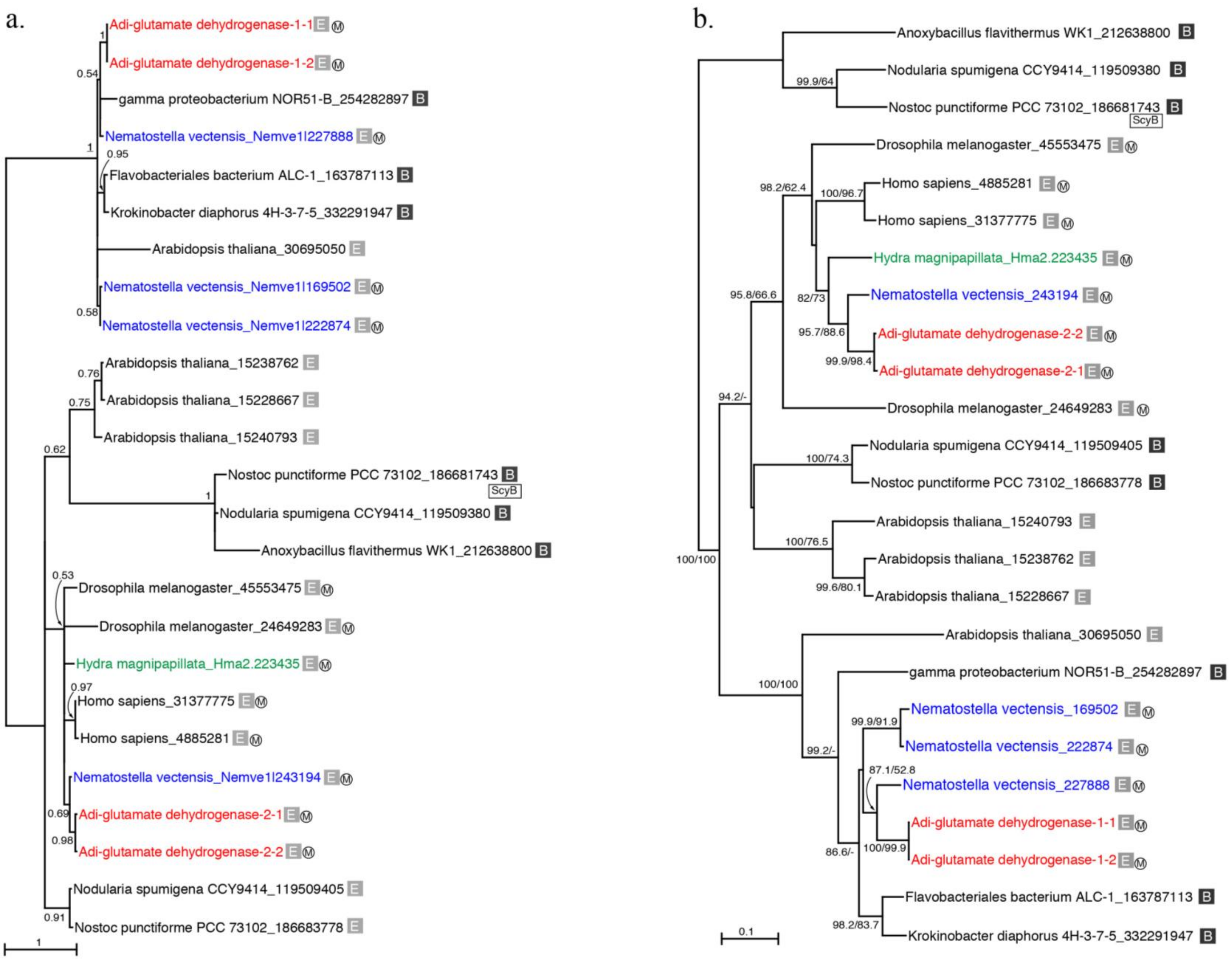
a.
Adi-glutamate dehydrogenase-1-1
Adi-glutamate dehydrogenase-1-2
gamma proteobacterium NOR51-B_254282897
Nematostella vectensis_Nemve1|227888
Flavobacteriales bacterium ALC-1_163787113
Krokinobacter diaphorus 4H-3-7-5_332291947
Arabidopsis thaliana_30695050
Nematostella vectensis_Nemve1|169502
Nematostella vectensis_Nemve1|222874
Arabidopsis thaliana_15238762
Arabidopsis thaliana_15228667
Arabidopsis thaliana_15240793
Nostoc punctiforme PCC 73102_186681743
ScyB
Nodularia spumigena CCY9414_119509380
Anoxybacillus flavithermus WK1_212638800
Drosophila melanogaster_45553475
Drosophila melanogaster_24649283
Hydra magnipapillata_Hma2.223435
Homo sapiens_31377775
Homo sapiens_4885281
Nematostella vectensis_Nemve1|243194
Adi-glutamate dehydrogenase-2-1
Adi-glutamate dehydrogenase-2-2
Nodularia spumigena CCY9414_119509405
Nostoc punctiforme PCC 73102_186683778
b.
Anoxybacillus flavithermus WK1_212638800
Nodularia spumigena CCY9414_119509380
Nostoc punctiforme PCC 73102_186681743
ScyB
Drosophila melanogaster_45553475
Homo sapiens_4885281
Homo sapiens_31377775
Hydra magnipapillata_Hma2.223435
Nematostella vectensis_243194
Adi-glutamate dehydrogenase-2-2
Adi-glutamate dehydrogenase-2-1
Drosophila melanogaster_24649283
Nodularia spumigena CCY9414_119509405
Nostoc punctiforme PCC 73102_186683778
Arabidopsis thaliana_15240793
Arabidopsis thaliana_15238762
Arabidopsis thaliana_15228667
Arabidopsis thaliana_30695050
gamma proteobacterium NOR51-B_254282897
Nematostella vectensis_169502
Nematostella vectensis_222874
Nematostella vectensis_227888
Adi-glutamate dehydrogenase-1-1
Adi-glutamate dehydrogenase-1-2
Flavobacteriales bacterium ALC-1_163787113
Krokinobacter diaphorus 4H-3-7-5_332291947
0.1

Figure S3. (**a**) BI molecular phylogeny of putative proteins from DSBA domain-containing genes. No *A. digitifera* ortholog to cyanobacterium DsbA (GI: 186681736) was found, although the *Nematostella* protein is more similar to cyanobacterium DsbA. Gene IDs, numbers, and scale bars are as in Figure S1a. The highlighted B indicates bacterial proteins; E indicates eukaryotic proteins; and a circled M denotes metazoan proteins. Enzymes of *A. digitifera* are shown in red, *Nematostella* proteins in blue, and *Hydra* in green. (**b**) NJ molecular phylogeny of putative proteins from DSBA domain-containing genes. No *A. digitifera* ortholog to cyanobacterium DsbA (GI: 186681736) was found; although the *Nematostella* protein is more similar to cyanobacterium DsbA. Gene IDs, numbers, and scale bars are as in Figure S1b.

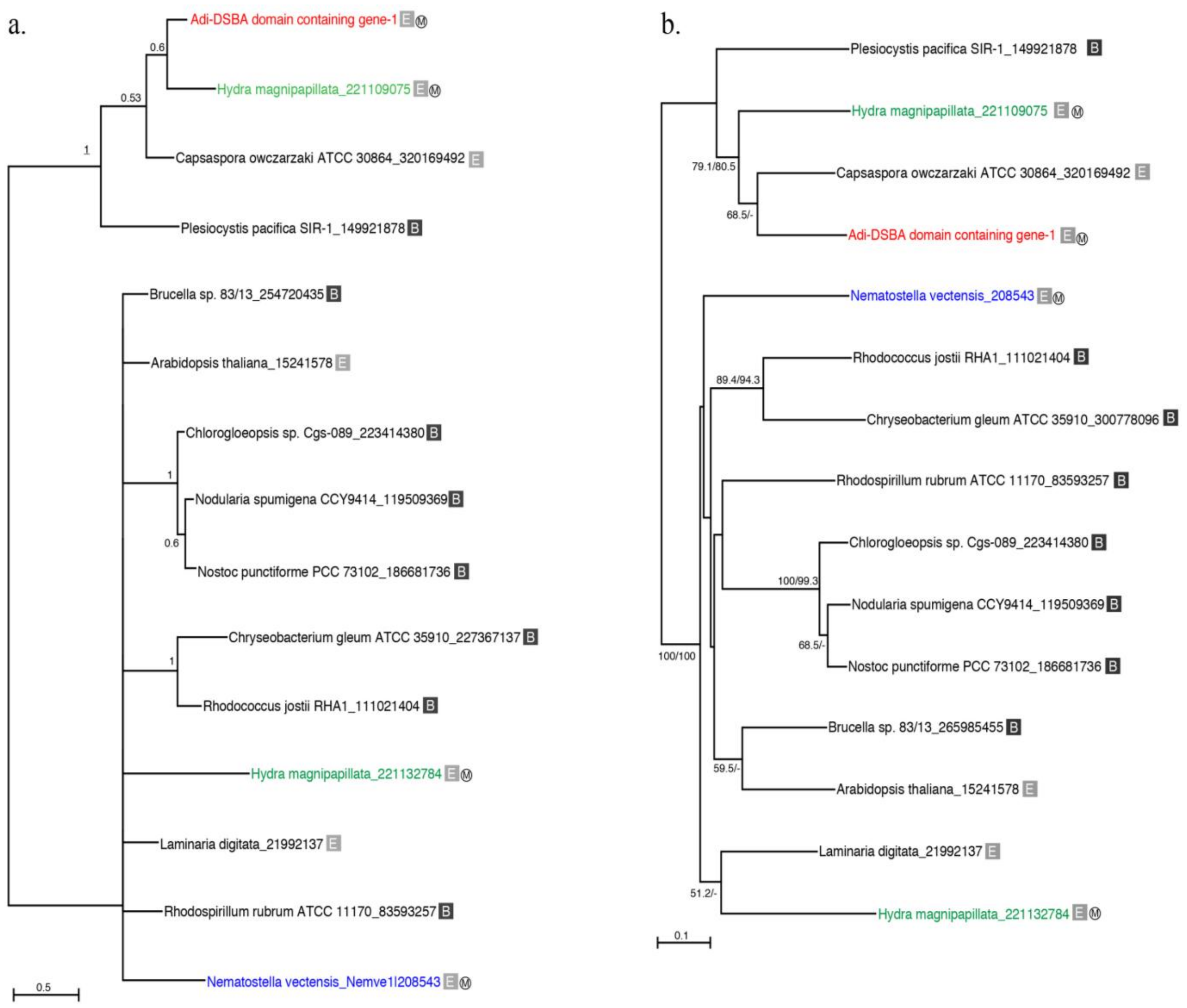

Figure S4. (**a**) Molecular phylogeny of TyrP, constructed by the BI method. The tree indicates the presence of two candidate genes for *tyrP* in the *Acropora* genome, although the *Nematostella* and *Hydra* genomes contain more candidate genes. *Acropora* TyrP1 is at a basal position among vertebrate tyrosinases. Details for gene ID, number, and scale bar are shown in Figure S1a. The highlighted B indicates bacterial proteins; E indicates eukaryotic proteins; and a circled M denotes metazoan proteins. Enzymes of *A. digitifera* are shown in red, *Nematostella* proteins in blue, and *Hydra* in green. (**b**) Molecular phylogeny of TyrP, constructed by the NJ method. The tree indicates the presence of two candidate genes for *tyrP* in the coral genome, although the *Nematostella* and *Hydra* genomes contain more candidate genes. Adi-TyrP1 is at a basal position among vertebrate tyrosinases. Details for gene ID, number, and scale bar are shown in Figure S1b.

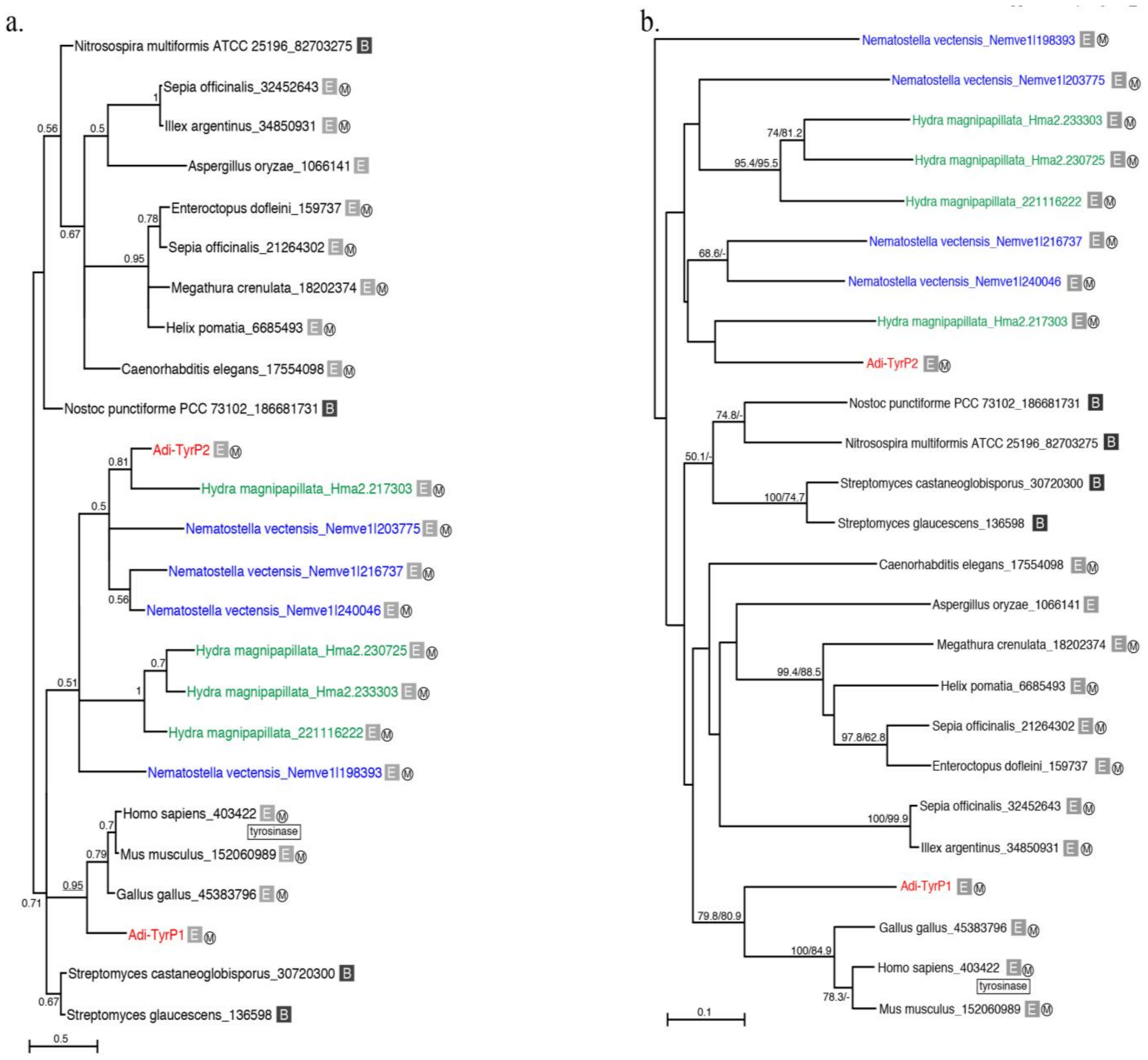

Figure S5. Molecular phylogenetic tree of PEP mutase, constructed by the NJ method. The tree indicates the presence of orthologous genes for *PEP mutase* in the coral genome. Details for gene ID, number, and scale bar are shown in Figure S1b.

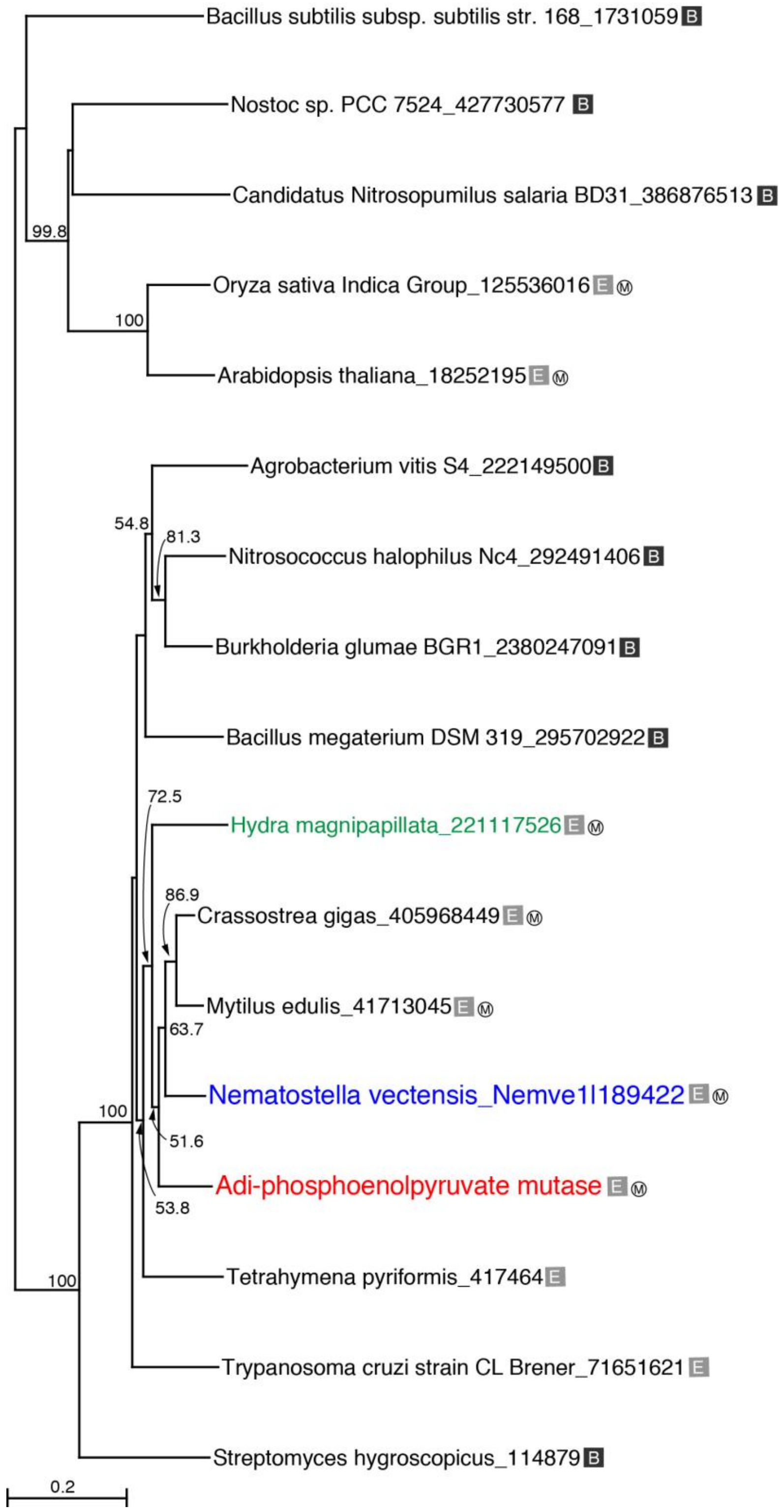

Figure S6. Molecular phylogenetic tree of Ppyr decarboxylase, constructed by the NJ method. The tree indicates the presence of orthologus genes for *Ppyr decarboxylase* in the coral genome. *Ppyr decarboxylase* orthologs were not found in *Drosophila*, *Homo*, and *Arabidopsis*. Details for gene ID, number, and scale bar are shown in Figure S1b.

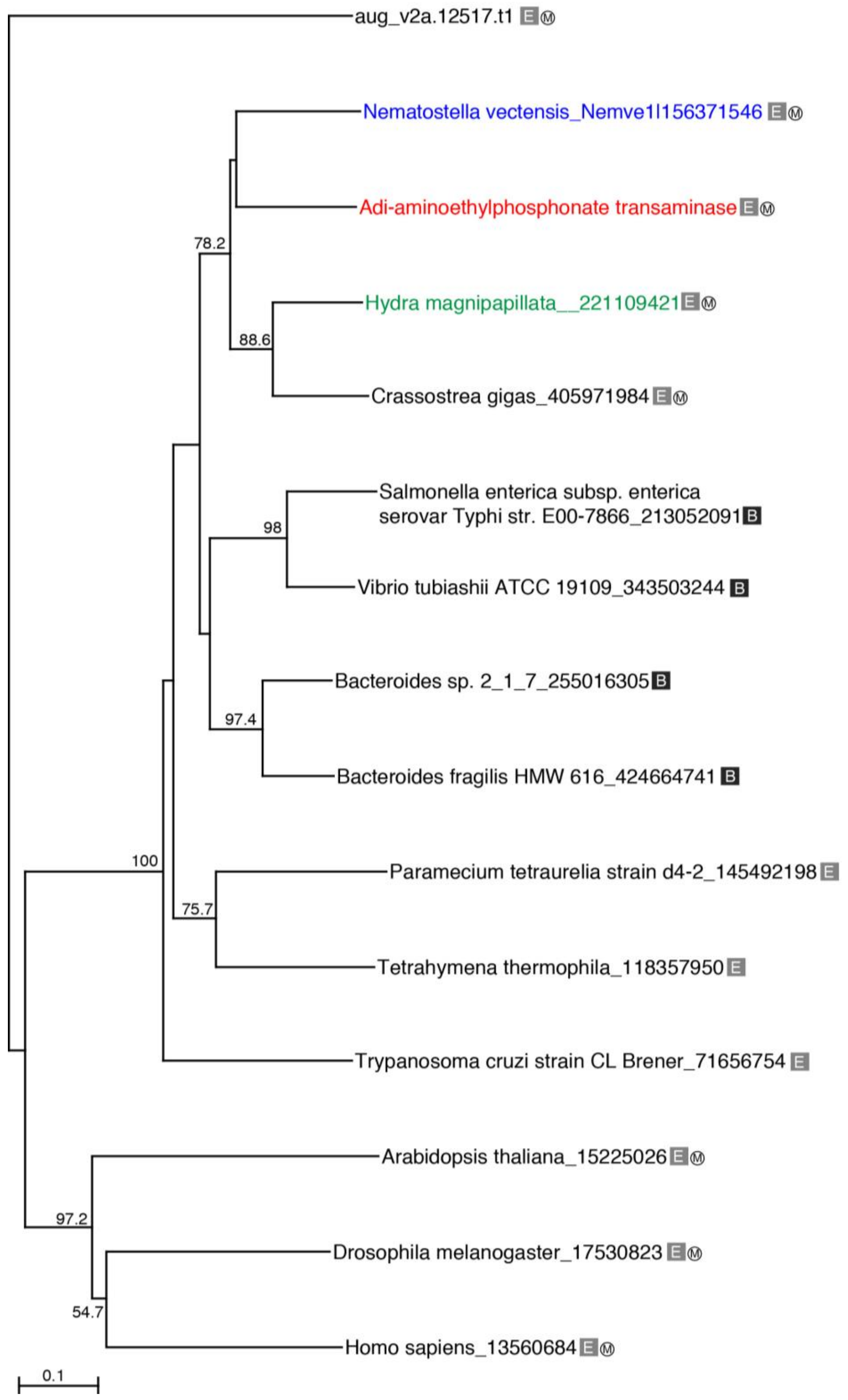

Supplement: Supplementary File 1 — Supplementary Information (PDF, 948 KB) [file marinedrugs-11-00559-s001.pdf]
